# Blast-Derived Small Extracellular Vesicles in the Plasma of Patients with Acute Myeloid Leukemia Predict Responses to Chemotherapy

**DOI:** 10.3390/biomedicines11123236

**Published:** 2023-12-07

**Authors:** Michael Boyiadzis, Chang-Sook Hong, Saigopalakrishna Yerneni, Annie Im, Brenda Diergaarde, Theresa L. Whiteside

**Affiliations:** 1 Department of Medicine, University of Pittsburgh School of Medicine, Pittsburgh, PA 15232, USA; 2 Department of Pathology, University of Pittsburgh School of Medicine Pittsburgh, PA 15232, USA; 3 UPMC Hillman Cancer Center, Pittsburgh, PA 15232, USA; 4 Department of Biomedical Engineering, Carnegie Mellon University, Pittsburgh, PA 15213, USA; 5 Department of Human Genetics, School of Public Health, University of Pittsburgh, Pittsburgh, PA 15232, USA

**Keywords:** acute myeloid leukemia, small extracellular vesicles (sEV), biomarker, bioprinting, leukemia-associated antigens, therapy responses

## Abstract

The small extracellular vesicles (sEV) accumulating in acute myeloid leukemia (AML) patients’ plasma are mixtures of vesicles produced by leukemic and non-malignant cells. sEV originating from leukemia blasts could serve as potential non-invasive biomarkers of AML response to therapy. To isolate blast-derived sEV from patients’ plasma, we developed a bioprinted microarray-based immunoassay using monoclonal antibodies (mAbs) specific for leukemia-associated antigens (LAAs) and mAbs specific for a mix of tetraspanins (CD9, CD63, and CD81). We determined the proportion of LAA^+^ sEV relative to total plasma sEV (the LAA^+^/total sEV ratio) in serially collected samples of newly diagnosed AML patients prior to, during, and after chemotherapy. At AML diagnosis, the LAA^+^/total sEV ratio was significantly higher in patients than in healthy donors (HDs). In patients who achieved complete remission (CR) after induction chemotherapy, the LAA^+^/total sEV ratios significantly decreased after each chemotherapy cycle to levels seen in HDs. In contrast, the LAA^+^/total sEV ratios in AML patients with persistent leukemia after therapy remained elevated during and after therapy, as did the percentage of leukemic blasts in these patients’ bone marrows. The LAA^+^/total sEV ratio emerges as a promising non-invasive biomarker of leukemia response to therapy.

## 1. Introduction

The recent emergence of small extracellular vesicles (sEV) in the plasma of cancer patients as potential biomarkers has introduced a new opportunity for seeking an alternative approach to monitoring responses to cancer therapies [1]. sEV are a subset of virus-sized (30–150 nm in diameter) vesicles that originate in the multivesicular bodies (MVBs) of parental cells by intra-vesicular membrane invaginations [2,3]. Upon fusion of MVBs with the cell membrane, sEV are released into the extracellular space. While sEV secretion occurs under physiologic conditions and all cells are capable of their release, tumor cells avidly produce sEV [2]. The sEV fractions obtained from the plasma of cancer patients are enriched with proteins expressed on the cell surface membranes and/or in the cytosol of the parent tumor cells. These attributes provide a rationale for considering plasma sEV as a surrogate for tumor cells. However, the sEV present in cancer patients’ plasma are mixtures of vesicles produced by all cells and originate from cancer as well as non-malignant cells [4]. Thus, in order to use tumor-derived sEV as markers of response to therapy, it is necessary to separate them from sEV derived from non-malignant cells. 

We and others have reported that patients newly diagnosed with acute myeloid leukemia (AML) have significantly higher levels of circulating sEV, measured as total sEV protein (TEP) levels, prior to any therapy compared to levels in healthy donors (HDs) [5,6,7]. Also, these levels remain elevated in some AML patients who achieve complete remission (CR) after induction chemotherapy [2,8]. We have also shown that leukemic blasts produce sEV rich in one or multiple leukemia-associated antigens (LAAs), while sEV produced by non-malignant cells carry few or no LAAs [9]. Based on these findings, we developed a novel, bioprinted microarray assay for immune capture and quantitation of sEV that carry LAAs (i.e., LAA^+^ sEV) in parallel to total sEV in the plasma of AML patients. Using this assay, the LAA^+^ sEV are captured using high-affinity monoclonal antibodies (mAbs) specific for four different LAAs overexpressed on leukemic blasts (CD123, CD117, CLL-1, and CD96), while total plasma sEV are captured using mAbs specific for tetraspanins that are carried by all sEV (CD9, CD63, and CD81).

Here, we describe the use of these custom microarrays for the capture of LAA^+^ and total sEV from plasma of AML patients obtained at diagnosis and serially during as well as post-chemotherapy. The quantitative capture in parallel microarray wells of LAA^+^ and total sEV fractions allowed for the determination of the LAA^+^/total sEV ratio. We show that the LAA^+^/total sEV ratios determined pre-, during, and post-chemotherapy in prospectively followed AML patients discriminated patients who achieved CR from those who had persistent leukemia following chemotherapy. 

## 2. Materials and Methods

### 2.1. Patients and Healthy Donors

For this study, we utilized data and blood collected at different time points from 28 newly diagnosed AML patients enrolled in a Phase II clinical trial conducted at our institution (NCT01829503) [10]. Blood was collected at the AML diagnosis prior to induction therapy and following therapy at the time when bone marrow biopsies were performed to evaluate the efficacy of the treatment. The clinical trial was designed for newly diagnosed AML patients who were not candidates for intensive induction chemotherapy. Induction therapy consisted of decitabine 20 mg/m^2^ IV daily on days 1–5, followed by a continuous infusion of cytarabine 100 mg/m^2^ on days 6–10. A bone marrow biopsy to assess early response was performed on day 15 after the initiation of induction therapy. If evidence of residual leukemia was observed (i.e., a biopsy showed >5% blasts), a second cycle of decitabine and cytarabine was administered. Patients who received a second cycle of induction therapy underwent a repeated bone marrow biopsy 15 days later. A bone marrow biopsy was also performed at the time of blood count recovery to assess the patient’s remission status; this occurred approximately 2 weeks after the bone marrow showed ≤5% blasts. Response assessments were determined using published criteria [11,12]. Blood was also collected from 11 healthy donors (HDs). All the patients and HDs provided written informed consent. The study was approved by the University of Pittsburgh Institutional Review Board. 

### 2.2. sEV Isolation

sEV were isolated from the plasma of AML patients and HDs and supernatants of leukemia cell lines (Kasumi-1, ThP1) using size-exclusion chromatography (SEC) performed on Sepharose 2B columns, preceded by differential centrifugation and ultrafiltration using 0.22 µm-pore Millipore filters, as previously described by us [13]. Pre-clarified plasma (1.0 mL) was placed on the SEC column and eluted with PBS in 1 mL fractions. sEV eluting in fraction #4 were harvested, and their protein content, size, nanoparticle numbers, morphology, and molecular content were determined, as previously described by us [6]. The protein content in fraction #4 was determined using the Pierce BCA protein assay kit (Pierce Biotechnology, Rockford, IL, USA), and the total sEV protein concentration was expressed as µg protein/mL of precleared plasma loaded onto the SEC column.

### 2.3. Western Blots

Western blots of sEV in fraction #4 were performed as previously described [14]. sEV were concentrated by centrifugation using a 100 K Amicon Ultra 0.5 mL centrifugal filter (EMD Millipore, Billerica, MA, USA) at 5000× *g*. sEV (10 µg protein) were loaded onto each lane of 7–15% SDS/PAGE gels, and after electrophoresis, they were transferred onto a PVDF membrane (Millipore, Billerica, MA, USA) and tested for the presence of sEV markers and LAAs using various Abs as previously described [14]. The following Abs were used: anti-CD96 (Abcam, Waltham, MA, USA, 56653, 1:500), anti-CD117 (Abcam 5506, 1:100 Biolegend), anti-TSG101 (Thermo Fisher, Waltham, MA, USA, PA5-31260, 1:500), anti-CD123 (R&D, Minneapolis, MN, USA, AF841, 1:1000), and anti-CLL-1 (R&D AF2946, 1:2000). Band intensities on exposed films were quantified using Image J software, Version 1.51 (NIH, USA). TSG101 was used as a marker of the sEV endocytic origin. The integrated pixel value was determined for each protein band by multiplying image intensity and band area after subtracting the mean background value.

### 2.4. Microarray Printing

Microarray printing was performed on a custom inkjet-based deposition system equipped with a 60 µm diameter diamond-like carbon-coated glass nozzle tip (MicroFab Technologies, Plano, TX, USA) as previously described [15]. The Ab bioinks were formulated in 100 mM sodium phosphate buffer with glycerol as a viscosity modulator. The concentration of Abs varied from 1 to 200 μg/mL, and the glycerol concentration was varied between 0 and 10% to find the optimal bioink for jetting reliability, defined as having a droplet velocity of ~2 m/s without satellite drop formation or nozzle clogging. The final concentration of 50 μg/mL protein was found to give the most reliable jetting and was therefore used for all the subsequent experiments. Jetted droplet formation and jetting stability were evaluated using a drop-in-flight JetXpert™ analysis system (Imagexpert, Inc., Nashua, NH, USA) for the different ink formulations. The microarray printing substrate consisted of 16-well Nexterion Slide H slides (75.6 mm × 25.0 mm, SCHOTT Nexterion^®^, DE, Louisville, KY, USA). 

### 2.5. Microarray: Antibodies

The Abs were printed as a cocktail mixture of 50 µg/mL of each Ab diluted in 100 mM sodium phosphate buffer with 2.5% glycerol. The following Abs were used for the capture of total plasma sEV: anti-CD9 (Biolegend, San Diego, CA, USA, HI9a), anti-CD63 (Biolegend, H5C6), and anti-CD81 (Biolegend, TAPA-11). The Abs used for the capture of LAA^+^ sEV consisted of anti-CD123 (Biolegend, 6H6), anti-CD117 (Biolegend, A3C6E2), anti-CD96 (Abcam, 81717), and anti-CLL-1 (Miltenyi, Gaitheraburg, MD, USA, 130-106-433). As positive and negative controls, 50 µg/mL of anti-human IgG and sodium phosphate buffer were used, respectively.

### 2.6. Microarray: Visualization and Data Analysis

After Ab printing, the dried slides were blocked with 1% bovine serum albumin at 4 °C overnight (~18 h). Samples, consisting of 5 µg of total plasma sEV protein (TEP), were diluted in 100 µL of washing buffer (PBS, 0.2% Tween^®^20), applied to the Ab cocktail for the capture of total sEV or of LAA^+^ sEV, and incubated under mild agitation for 1 h at room temperature (RT), followed by stationary overnight incubation (~18 h) at 4 °C. Following sample incubation, the slides were washed three times (15 min each) in washing buffer. A cocktail of detection Abs diluted in wash buffer 1:500 (anti-CD9, CD63 and CD81) tagged with Alexafluor 647^®^ (eBioscience, San Diego, CA, USA) using NHS ester chemistry was applied, and the slides were incubated under mild agitation for 2 h at RT. The slides were then washed three times (15 min each) with wash buffer, followed by washes ×3 (15 min each) with deionized water (18 Ohm). Images were acquired using a Zeiss LSM 880 confocal microscope (Carl Zeiss Microscopy, Thornwood, NY, USA) and the tiling feature under constant imaging settings across all the samples. Analysis was performed using Zen Blue v2.1 image analysis software (Carl Zeiss microscopy, Thornwood, NY, USA) by drawing a region-of-interest bounding each array consisting of 36 individual spots. For every sample, the ratio of the fluorescence intensity of the LAA^+^ sEV fraction to the total plasma sEV fraction was calculated after background correction for comparative analysis. 

### 2.7. Microarray: Optimization for Immunocapture of LAA^+^ and Total Plasma sEV 

The principle of the bioprinted microarray-based assay for immune capture and detection of sEV that carry LAAs (LAA^+^ sEV) in parallel to total sEV is shown in Supplementary Figure S1. To establish the microarray platform, experiments were first performed to test the binding and detection characteristics of the individual mAbs selected for immune capture of LAA^+^ sEV. Each of the four mAbs was individually bioprinted on a microarray at increasing concentrations. Each LAA-specific capture mAb and a cocktail of capture Abs specific for tetraspanins (CD63, CD9, and CD81) were printed in parallel as an array of 36 identical spots, using 10–60 overprints (OPs). The optimal concentration of LAA-specific mAb was defined as the number of OPs used, with an OP defined as the number of drops of each mAb delivered to each of the 36 spots/square. Supplementary Figure S2 shows representative titration results based on differences in mAb OPs used for sEV binding to each mAb. The optimal printed amount of mAb was determined to be 40 OPs over an area of 500 µm^2^, resulting in a final concentration of 86 ng of mAb protein per microarray pattern (density of mAb = 0.172 ng/µm^2^).

### 2.8. Statistical Analyses

Wilcoxon–Mann–Whitney tests were used to evaluate differences between different subject groups (e.g., patients vs. HDs and CR patients vs. PD patients). The Friedman test was used to determine if the LAA^+^/total sEV ratios at the different timepoints were significantly different from each other. Wilcoxon signed-rank tests were used to evaluate differences in the LAA^+^/total sEV ratios and other characteristics between two timepoints. *p* values < 0.05 were considered significant. Statistical analyses were performed using the SAS^®^ statistical software package (SAS version 9.4, SAS Institute Inc., Cary, NC, USA).

## 3. Results

### 3.1. Patient Characteristics

We utilized blood collected from 28 patients newly diagnosed with AML prior to any therapy as well as serially during and after therapy. Demographic and baseline characteristics are presented in Table 1. The mean age at diagnosis was 75.5 years, and 32.1% of the patients were female. Twenty (71.4%) patients achieved complete response (CR) or CRi (CR with incomplete count recovery) after two cycles of induction chemotherapy, while eight (28.6%) patients had persistent disease (PD) after two cycles of induction chemotherapy.

### 3.2. Characterization of sEV Isolated from AML Patients’ Plasma

**Figure 1:**
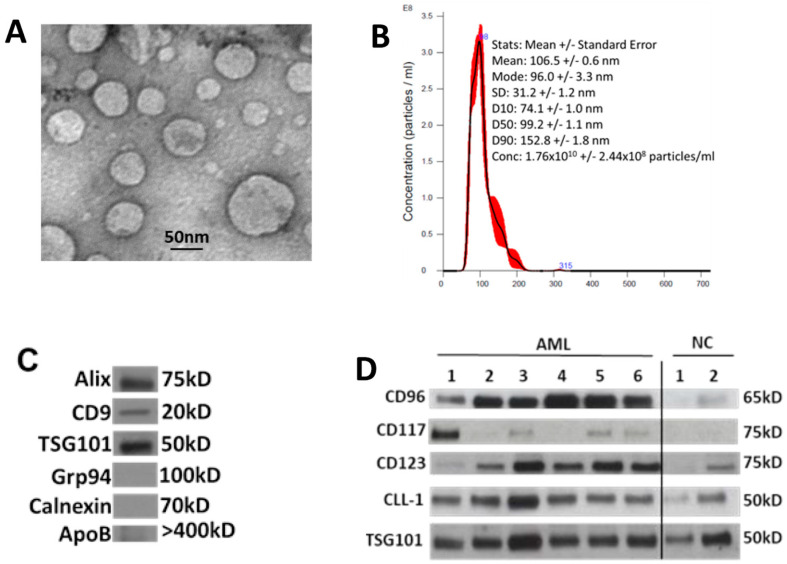
Characteristics of sEV isolated from AML patients’ plasma at diagnosis: (**A**) transmission electron microscopy (TEM) of isolated sEV, (**B**) size and concentration of sEV as determined by NanoSight, (**C**) Western blots show that sEV carry endocytic markers (Alix, CD9, and TSG101) but not cytoplasmic proteins such as Grp94 or Calnexin, and (**D**) Western blots show the presence of LAAs in the isolated sEV. LAAs are differentially expressed in each patient’s sEV. Each lane was loaded with 10 μg of sEV protein. TSG101 is a marker that confirms the endocytic origin of sEV. NC: sEV isolated from healthy donors’ plasma.

Figure 1 illustrates features of sEV evaluated according to the criteria recommended by the ISEV [16]. The vesicles isolated from AML patients’ plasma or AML cell line supernatants fit into the category of sEV [16]. Transmission electron microscopy (TEM) shows the presence of vesicles ranging in size from 30 to 150 nm, as also confirmed by NanoSight (Figure 1A,B). Western blots indicate that these vesicles carry endocytic markers (Alix, TSG101) and do not carry cytosolic proteins (Grp94, Calnexin) or ApoB (Figure 1C). Further, sEV isolated from the plasma of AML patients carried at least one or more of the four evaluated LAAs (CD123, CD117, CLL-1, and CD96) (Figure 1D).

### 3.3. Total sEV Protein (TEP) Levels in AML Patients’ Plasma 

**Figure 2:**
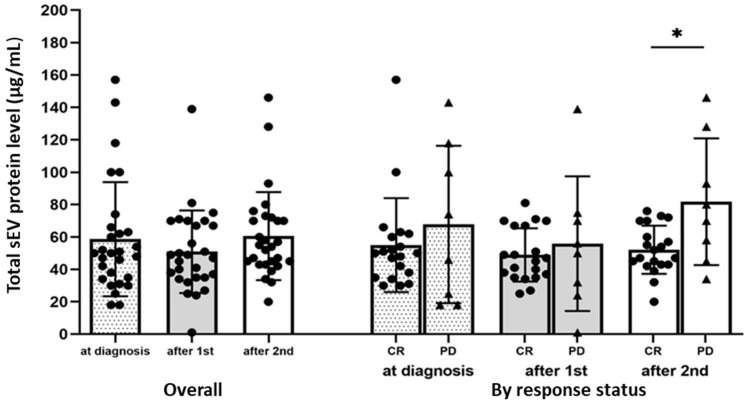
Total sEV protein (TEP) levels in AML patients’ plasma pre-, during, and post-chemotherapy. TEP levels at diagnosis after the first and second chemotherapy cycles are shown overall (N = 28) and stratified by response (CR: patients who achieved complete remission (including those with CRi) (N = 20); PD: patients with persistent disease, N = 8). * *p* = 0.05, Wilcoxon–Mann–Whitney test.

TEP levels at AML diagnosis and after the first and second cycles of chemotherapy did not differ significantly (mean ± sd (range), 58.6 ± 35.2 (18–157), 50.9 ± 25.5 (1–139), and 60.6 ± 27.2 (20–146), respectively) (Figure 2). In addition, TEP levels at diagnosis and after the first cycle of chemotherapy did not differ significantly between those who would achieve CR and those who had PD. The difference in TEP levels after the second cycle of chemotherapy was borderline significant (*p* = 0.0499; 52.1 ± 14.8 (20–76) vs. 81.8 ± 39.2 (34–146) (Figure 2). 

### 3.4. LAA^+^ sEV in AML Patients’ Plasma Measured by Microarrays

Because TEP levels did not clearly distinguish patients who would achieve CR from those with PD, we developed a bioprinted microarray-based assay for immune capture and quantitation of sEV that carry LAAs (i.e., LAA^+^ sEV) in parallel to total sEV in the plasma of AML patients. 

Spiking experiments (Supplementary Figures S3 and S4) were performed to demonstrate that the microarray assay can discriminate between leukemic sEV and non-leukemic sEV in AML plasma. These experiments showed that the microarray assay discriminated leukemic (LAA^+^) sEV from non-leukemic sEV at a protein concentration of 1 µg/mL. At the protein concentration of 5 µg/mL of non-malignant cell-derived sEV, the LAA^+^/total sEV ratio was only 0.12. In contrast, the LAA^+^/total sEV ratio was 0.95 for 5 µg/mL of LAA^+^ Kasumi (leukemia) cell-derived sEV (Supplementary Figure S3). These results suggest that the microarray platform has adequate sensitivity and specificity for the detection of lower sEV levels recovered from the plasma of HDs.

Using this assay, we determined the LAA^+^/total sEV ratio in the serially collected plasma samples from 16/27 AML patients for whom plasma was still available. Of these 16 patients, 9 achieved CR and 7 had PD after two cycles of chemotherapy. 

**Figure 3:**
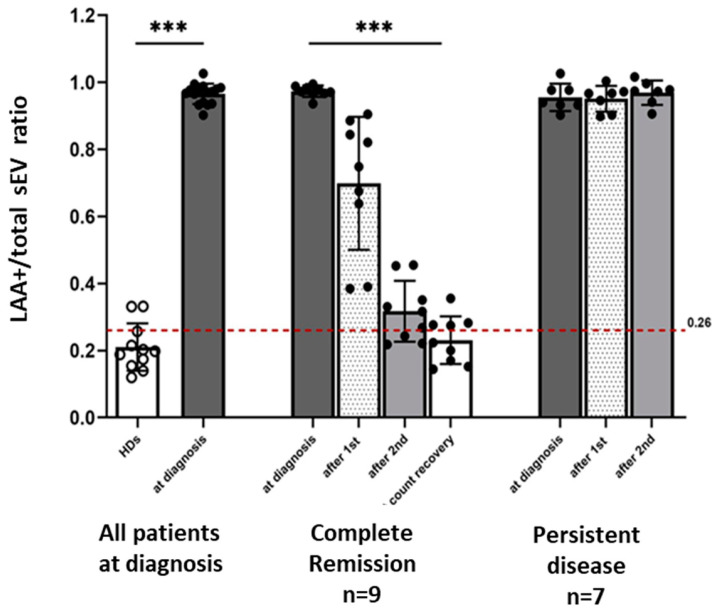
The LAA^+^/total sEV ratio and response to leukemia therapy. At AML diagnosis prior to therapy, the LAA^+^/total sEV ratio was significantly higher in all patients (N = 16) compared to the LAA^+^/total sEV ratio of HDs (N = 11). In patients who achieved CR (N = 9) after therapy, there was a decrease in the LAA^+^/total sEV ratios after each cycle of therapy, paralleling the decrease in the bone marrow blasts. In patients who had persistent leukemia (N = 7) after therapy, the ratios of LAA^+^/total plasma sEV remained elevated even after 2 cycles of induction chemotherapy. *** *p* < 0.0001; the Wilcoxon–Mann–Whitney test was used to compare patients with healthy donors (HDs); the Friedman test was used to compare the ratios between the different timepoints, separately for the CR and PD groups. The cut-off used to determine the sensitivity and specificity of the ratio to discriminate AML patients at diagnosis from HDs was 0.26.

At AML diagnosis prior to therapy, the LAA^+^/total sEV ratio was significantly higher in patients compared to HDs (Figure 3). The mean ratio at diagnosis was 0.97 ± 0.03 vs. 0.21 ± 0.07 for HDs (*p* < 0.0001). Using a confidence interval of 95%, the cut-off for the LAA^+^/total sEV ratio was set at 0.26. Applying this cut-off to discriminate patients at diagnosis from HDs, the LAA^+^ /total sEV ratio had 100% sensitivity and 81.8% specificity. In patients who achieved CR after receiving induction chemotherapy, the LAA^+^/total sEV ratios decreased after each cycle of chemotherapy, reaching a ratio similar to that of HDs (AML patients vs. HDs: after 2 cycles, 0.32 ± 0.09 vs. 0.21 ± 0.07, *p* = 0.01; at count recovery, 0.23 ± 0.07 vs. 0.21 ± 0.07, *p* = 0.49; Figure 3). In parallel with the decrease in the LAA^+^/total sEV ratio, percentage blasts in the bone marrow biopsy also decreased significantly in the patients who achieved CR (42.0% blasts at AML diagnosis vs. 2.8% after the second cycle of chemotherapy, *p* = 0.004). In contrast, the LAA^+^/total sEV ratios in the plasma of AML patients who had persistent leukemia after therapy did not change (*p* = 0.65) and remained elevated during and after therapy (Figure 3; after cycle 1 and after cycle 2, respectively: 0.95 ± 0.04 and 0.97 ± 0.04, *p* < 0.0001 compared to HDs for both), as did their percentage bone marrow blasts (60.3% blasts at AML diagnosis vs. 52.0% after the second cycle of chemotherapy, *p* = 0.58). Supplementary Figure S5 shows the individual microarray results for each of the 16 AML patients and the 11 HDs.

## 4. Discussion 

The recent emergence of sEV as potential cancer biomarkers has provided a rationale for the development of methods that would provide for reliable non-invasive monitoring of cancer patients and evaluations of responses to oncologic therapies. In patients with solid tumors, total protein levels of sEV isolated from cancer patients’ plasma were reported to be predictive of response to therapy [17]. As sEV are known to be abundantly produced by stressed cells, including leukemia blasts, total plasma sEV levels in AML patients were expected to correlate with response to therapy. However, we found that the total sEV protein (TEP) levels did not predict response to therapy in AML patients. In fact, TEP levels remained elevated throughout sequential cycles of chemotherapy in patients who achieved CR and in patients with PD. There are several potential explanations for this finding. It is possible that chemotherapy increased sEV production levels by non-malignant cells. It is also possible that increased levels of sEV in the plasma of CR patients originated from residual leukemia cells or from recovering mononuclear cells. Thus, in contrast to solid cancers, where TEP levels decline in patients that respond to chemotherapy, TEP levels in AML plasma did not correlate with response to therapy. In the current study, we isolated sEV using size-exclusion chromatography [13]. sEV in early fraction #4 contains largely non-aggregated, morphologically intact vesicles relatively free of contaminating plasma proteins (IgG, albumins, and other plasma proteins, which elute in later fractions); thus, although not pure, the isolated sEVs are in part depleted of plasma proteins. This potentially could have affected the measured total sEV protein levels.

We considered the possibility that sEV derived from leukemic blasts, which carry high levels of LAAs, correlate better with response to therapy than total plasma sEV. To this end, we developed an immune capture-based method for the isolation and quantification of LAA^+^ sEV from AML patients’ plasma independently of total plasma sEV. The latter were captured using mAbs specific for tetraspanins that are carried by most sEV. The LAA^+^ sEV were captured from the same plasma sample using a mix of high-affinity mAbs specific for four different LAAs. The method effectively separated total sEV from LAA^+^ sEV in the same plasma sample, allowing for the determination of the LAA^+^/total sEV ratio for each AML patient. This ratio could then be linked to the patient’s response to induction chemotherapy. The data we report show that by separating sEV into two subsets by immune capture and calculating their ratio, it is possible to predict the responses of AML patients to chemotherapy.

The success of immunocapture of blast-derived sEV from AML plasma is strictly dependent on the selection of mAbs specific for LAAs. Because no individual mAb specific for AML blasts exists, a cocktail of mAbs specific for four different LAA antigens overexpressed on leukemic blasts and carried by blast-derived sEV was used to create the immune capture platform. The high-affinity interleukin-3 receptor α chain (IL-3Rα and CD123) is reported to be present in >80 % of blasts, the receptor for proto-oncogene c-Kit (CD117) in 74% of blasts, the C-type lectin molecule-1 (CLL-1) in 92% of blasts, and the type I membrane immunoglobulin CD96 in 75% of blasts [18,19,20,21,22,23,24,25,26]. Importantly, we have determined that these antigens are carried by sEV produced by primary AML blasts, as measured by Western blots. We hypothesized that a cocktail of mAbs specific for each of these four LAAs used for immune capture of sEV in AML plasma would be more effective than any single LAA for selective capture of blast-derived sEV. In fact, the immunocapture microarray assay we have established consistently detected the existence of LAA^+^ sEV in AML plasma, which was presumably derived from leukemia cells in the bone marrow. Further, the proportion of LAA^+^ sEV relative to total sEV in plasma appears to be a reliable measure of the presence and activity of leukemia blasts that undergo changes during chemotherapy. The LAA^+^/total sEV ratios in the plasma of AML patients were significantly elevated at diagnosis relative to sEV in HDs’ plasma, emphasizing the diagnostic potential of the ratio. The prognostic potential of the ratio was also promising; the LAA^+^/total sEV ratios consistently and significantly decreased in the post-induction chemotherapy plasma of patients who responded to chemotherapy and achieved CR. In contrast, it remained elevated in AML patients with persistent leukemia after therapy. 

A bone marrow biopsy is the de facto standard for evaluating an AML patient’s response to therapy and is often repeated multiple times during treatment. Results of bone marrow biopsies following therapy are important, as they guide the treatment plan [27]. When performed by trained personnel, the procedure is safe, but it remains uncomfortable and can be associated with pain and, less often, with bleeding and infections [28,29,30,31,32,33,34,35,36]. In children with AML and in some adult patients with AML, sedation/anesthesia is required for performing the biopsy, and there is always the possibility that the biopsy specimen is not adequate for evaluation [37,38,39]. Performing a bone marrow biopsy in some AML patients with hemodynamic instability may not be feasible. The future use of this assay in serial monitoring of the LAA^+^/total sEV ratios in the plasma of AML patients may provide a non-invasive, sensitive platform for detecting therapy-related changes in leukemic blasts that could guide chemotherapy selection, reduce the frequency of repeated bone marrow sampling, and improve the quality of life for AML patients.

Currently, assessments of patients’ responses to therapy are based on morphological evaluations of the presence/absence of blasts in the bone marrow and on multicolor flow cytometry results. In this study, we did not evaluate CR patients for the presence/absence of measurable residual disease (MRD). However, we expect that differences in the LAA^+^/total sEV ratios between the MRD-negative and MRD-positive CR patients will be distinct. We expect the ratios in MRD-negative CR patients to be similar to the low ratios observed in HDs. On the other hand, MRD-positive CR patients are expected to have higher ratios, as they have residual blasts in the bone marrow actively producing blast-derived LAA^+^ sEV. Further studies are needed to verify the role of the LAA^+^/total sEV ratios in MRD assessments.

Liquid biopsies are increasingly being used for cancer molecular profiling and have emerged as promising non-invasive biomarkers. In AML, next-generation sequencing (NGS) of circulating cell-free DNA (ccfDNA) has been shown to identify mutations that are not detected by bone marrow sequencing. Furthermore, sequential sampling of the ccfDNA of patients in remission also identified patients with new or persistent mutations that appeared to signal an impending relapse [40]. In MDS, serial monitoring of cell-free circulating tumor DNA allowed concurrent tracking of both mutations and karyotypic abnormalities throughout treatment and enabled the prediction of responses to therapy [41]. Patients with AML or myelodysplastic syndromes with persistent circulating tumor DNA status either at 1 month or 3 months after allogeneic hematopoietic cell transplantation had a significantly higher risk of relapse and death than those with negative status [42]. Furthermore, increasing ctDNA levels between 1 month and 3 months post-transplant was the precise predictor of relapse [42]. Similarly, blast-derived sEV analysis could be investigated for predicting leukemia relapse, evaluation of clonal dynamics, and mechanisms of therapy resistance.

## 5. Conclusions

Our data show that a high LAA^+^/total sEV ratio after chemotherapy is associated with leukemia persistence, whereas a low ratio is associated with response to therapy. The future use of this assay in serial monitoring of the LAA^+^/total sEV ratios in the plasma of AML patients provides a non-invasive, sensitive platform for detecting therapy-related changes in leukemic blasts that could guide chemotherapy selection, reduce the frequency of repeated bone marrow sampling, and improve the quality of life for AML patients.

## Figures and Tables

**Table 1:** Patients’ characteristics at AML diagnosis.

Characteristic	N = 28
Age in years, mean (range)	75.5 (68–83)
Sex, N (%) female	9 (32.1)
Cytogenetic risk category, N (%)	
Favorable	1 (3.6)
Intermediate	12 (42.9)
Unfavorable	14 (50.0)
Unknown	1 (3.6)
WBC ^a^ count (×10^9^/L), mean (range)	11.4 (0.8–54.8)
Platelet count (×10^9^/L), mean (range)	77.5 (16–354)
% blasts in bone marrow, mean (range)	47.2 (8–94)
% blasts in peripheral blood, mean (range)	18.6 (0–81)

^a^ WBC: white blood cell.

## Data Availability

The data presented in this study are available on request from the corresponding author and institutional approval.

## Supplementary Materials

The following supporting information can be downloaded at: https://www.mdpi.com/article/10.3390/biomedicines11123236/s1, Figure S1: Schematic for microarray-based immune capture and detection of plasma-derived sEV; Figure S2: Titrations of mABs for immunocapture; Figure S3: Quantitation and detection of LAA^+^ and total sEV—spiking experiments; Figure S4: Microarray assay: sensitivity of detection; Figure S5: Microarray-based immune capture and detection of sEV in plasma from AML patients and healthy donors.
