# Supplementary material for: Blast-Derived Small Extracellular Vesicles in the Plasma of Patients with Acute Myeloid Leukemia Predict Responses to Chemotherapy
Source: Biomedicines. 2023 Dec 7;11(12):3236. doi: 10.3390/biomedicines11123236 (PMC10740822; doi:10.3390/biomedicines11123236)
Supplement: Supplementary file 1 [file biomedicines-11-03236-s001.zip › biomedicines-2732088-supplementary.pdf]

Supplemental Figure S1

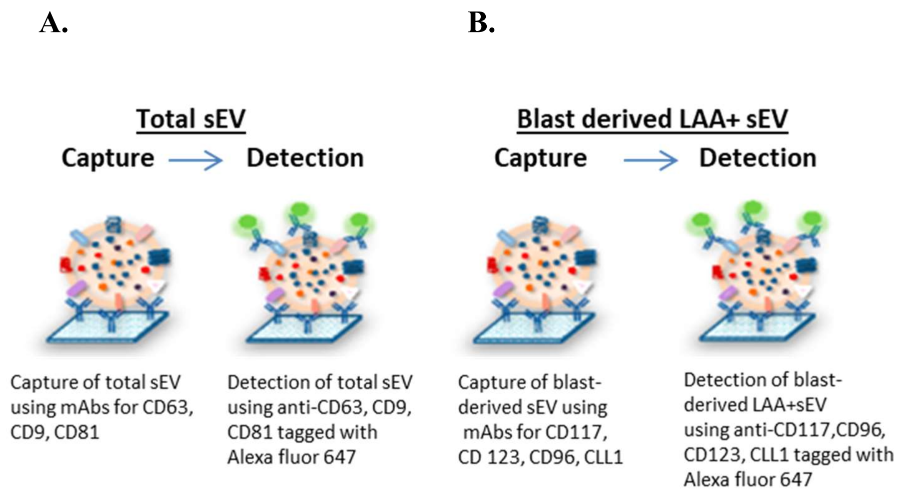

Supplemental Figure S1. Schematic for microarray-based immune capture and detection of plasma-derived sEV. (A) Total sEV are captured by a mix of bioprinted tetraspanin-specific mAbs (CD63, CD9, and CD81). The detection of total sEV is accomplished with fluorescently labeled anti-tetraspanin mAbs. **(B)** A cocktail of four bioprinted mAbs recognizing LAAs (CD117, CD123, CD96, and CLL1) is used for the capture of blast-derived LAA$^+$ sEV. Detection of LAA$^+$ sEV is accomplished with fluorescently labeled anti-tetraspanin mAbs. The A and B microarrays are tested in parallel to assess the relative fluorescence intensity (RFI) of each sample. Data obtained from the A and B microarrays are used to calculate the LAA$^+$/total sEV ratio in each sample.

Supplemental Figure S2.

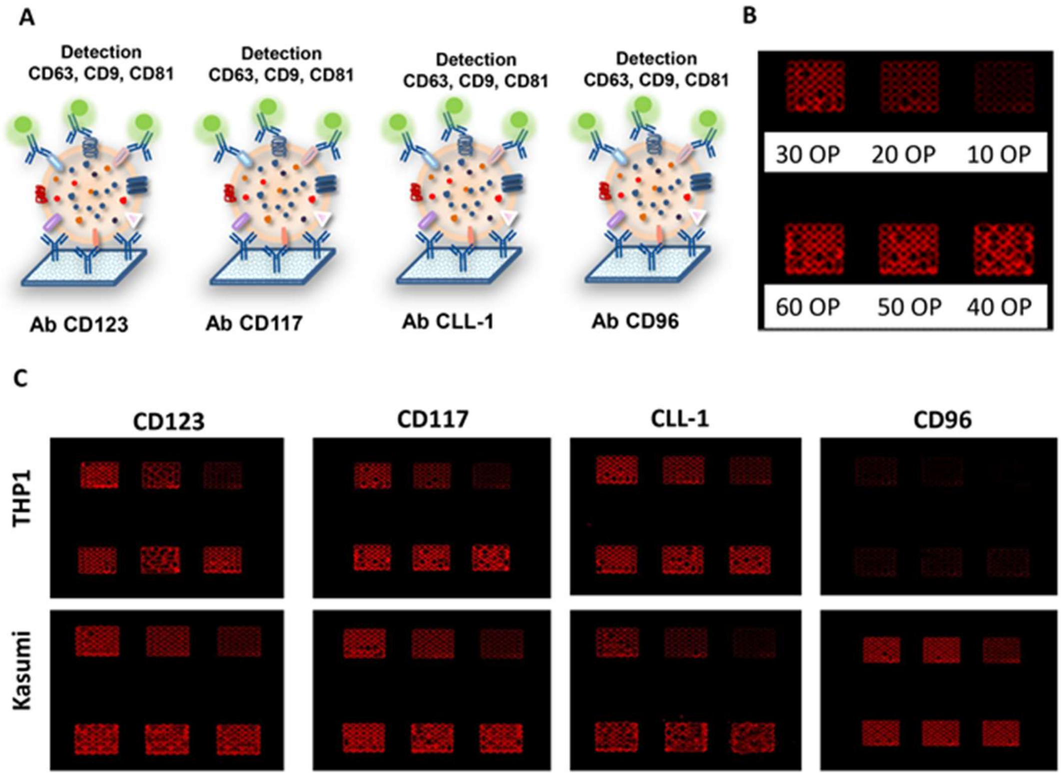

Supplemental Figure S2. Titrations of mABs for immunocapture. In **A,** the schema for titrations of *individual* anti-LAA mAbs used for the capture and detection of LAA^{+} sEV. The mAbs specific for these LAAs were deposited on microarrays and used for establishing optimal Ab dilutions. In **B**, arrays were printed with increasing doses of mAb, as indicated by the numbers of the applied overprints (OP), which ranged from 10 to 60 OPs. In **C**, real-life images of the microarrays illustrate dilutions of mAbs (OPs #) that are optimal for sEV capture from THP-1 (monocytic cell line) or Kasumi (leukemia cell line) supernatants.

Supplemental Figure S3.

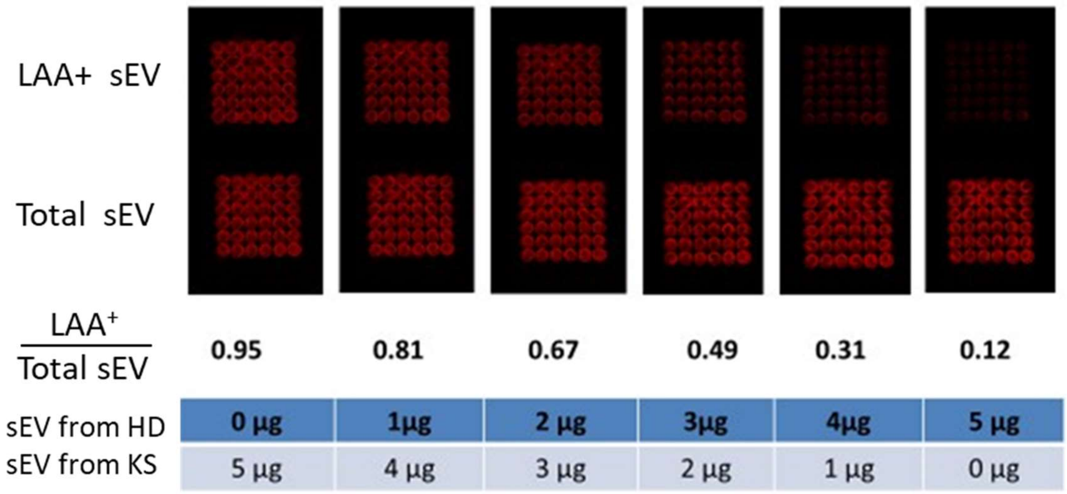

Supplemental Figure S3. Quantitation and detection of LAA$^+$ and total sEV – spiking experiments. sEV isolated from the plasma of healthy donors (HDs) were mixed with sEV isolated from Kasumi cells (KS) at increasing/decreasing concentrations. Mixed sEV were plated in microwells and immunocaptured with respective mAbs, as illustrated in Supplemental Figure 1, and confocal microscopy was used for the detection of LAA$^+$ sEV (upper row) and total plasma sEV (lower row). The LAA$^+$/total sEV ratios were calculated and are shown below the microarray. The ratios decreased as the concentrations of LAA$^+$ sEV (from Kasumi cells) decreased. As expected, HDs sEV gave only a minimal detection signal for LAAs at a ratio of 0.12.

Supplemental Figure S4.

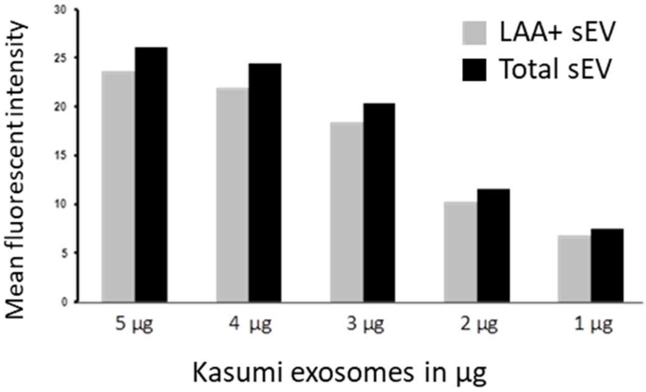

Supplemental Figure S4. Microarray assay: sensitivity of detection. The sensitivity of detection was determined by testing decreasing concentrations (5 µg/mL – 1 µg/mL) of Kasumi sEV and measuring the detectable signal (mean fluorescent intensity, MFI). At 1 µg/mL Kasumi sEV, the MFI was 6, indicating that the lower limit of detection of the microarray is 1 µg total sEV protein.

Supplemental Figure S5.

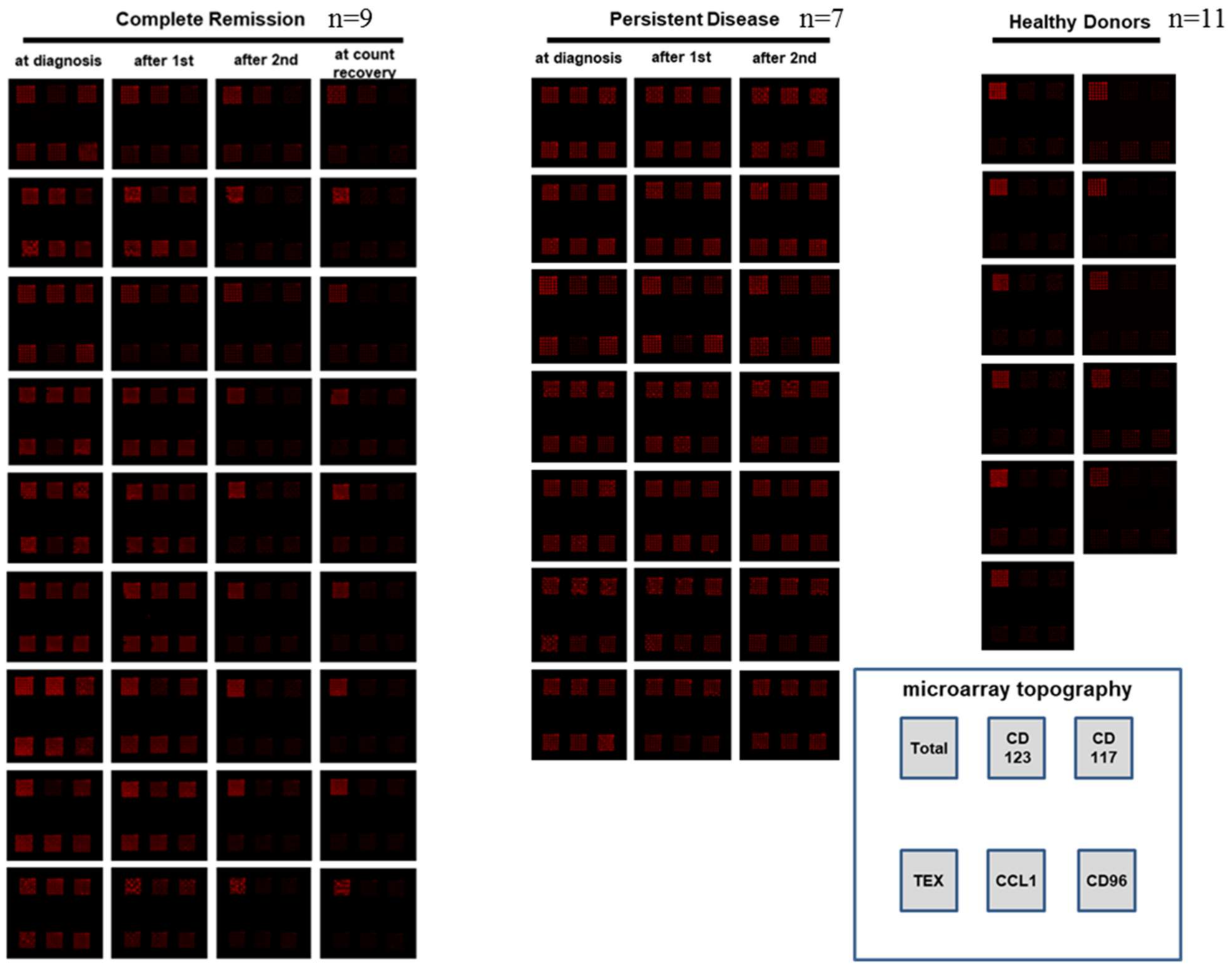

Supplemental Figure S5. Microarray-based immune capture and detection of sEV in plasma from AML patients and healthy donors. The schema (right) shows the microarray topography for each square and the capture of total or LAA^{+} sEV by the indicated individual monoclonal antibodies (mAbs) bioprinted on the array (right 4 squares), total sEV (cocktail of anti-CD63, anti-CD9, and anti-CD81mAbs), and LAAs^{+} sEV (cocktail of anti-CD123, anti-

CD117, anti-CLL-1, and anti-CD96). Scans of the microarrays are shown for the 16 patients (9 who achieved CR and 7 with PD) and 11 HDs. In patients who achieved CR, there was a decrease in the LAA$^+$/total ratios after each cycle of therapy; the ratios of LAA$^+$/total plasma sEV remained elevated even after 2 cycles of induction chemotherapy in those with PD. Note that in HD's samples, only total sEV squares are positive. The figure also shows that the use of high-affinity mAbs specific for four different LAAs overexpressed on leukemic blasts (CD123, CD117, CLL-1, and CD96) increases the sensitivity of the assay for LAA$^+$ sEV.
